# Book Reviews

**DOI:** 10.1556/JBA.4.2015.1.8

**Published:** 2015-03-18

**Authors:** Csilla Ágoston

**Affiliations:** Doctoral School of Psychology Department of Clinical Psychology and Addiction Eötvös Loránd UniversityBudapestHungaryagoston.csilla@ppk.elte.hu

**Figure:**
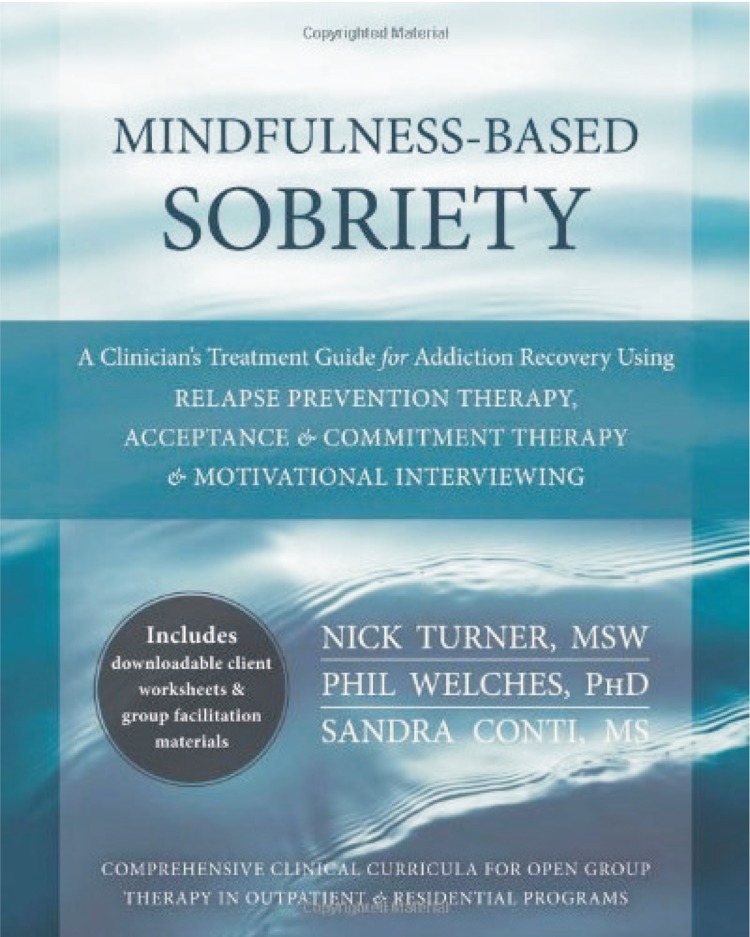


In the last few years, mindfulness-based therapies have reached a spectacularly increasing popularity, several new books in the topic are being published year after year. There is now a lot of evidence for the effectiveness of different kinds of mindfulness-based therapies in the treatment of addictions (e.g. Hayes & Levin, 2012), and numerous self-help books (e.g. Williams & Craft, 2012) have also been published in the topic. So why is this current book special or more than the former ones?

The *Mindfulness-based sobriety: A clinician’s treatment guide for addiction recovery using relapse prevention therapy, acceptance and commitment therapy, and motivational interviewing *introduces a comprehensive approach for the treatment of different kinds of addictions; as the title declares, the framework of mindfulness-based sobriety (MBS) incorporates the elements of acceptance and commitment therapy (ACT), relapse prevention therapy (RPT) and motivational interviewing (MI). MBS is an integrated approach because it does not require a shift from one model to another, but has a single, unifying process. Mark Twain once said that “giving up smoking is the easiest thing in the world. I know because I’ve done it thousands of times.” This somewhat sarcastic quote highlights the fact that action cannot be easily converted to sustainment and draws attention to the problem of lapses and relapses which are very common during the treatment of addictions. MBS pays attention to the discussion of lapses and relapses with patients and focuses on maintaining sobriety through the enhancement of awareness and exploration of individuals’ values.

The authors are very experienced clinicians and experts of the field. The first author, Nick Turner, is a clinical supervisor, a licensed clinical social worker (LCSW) and a certified alcohol and drug counselor (CADC). He is currently working at Gateway Foundation, in Chicago, IL and at the University of Chicago. Turner has worked in both outpatient and residential mental health and substance abuse settings with children, adolescents, and adults. Phil Welches, PhD, is a clinical psychologist and the clinical director for Gateway Foundation’s community services division. He is the past director of two non-profit addiction treatment centers and he provides therapy for substance abuse and mental health problems using a collaborative mindfulness-based approach. They are members of the Association for Contextual Behavioral Science and the Motivational Interviewing Network of Trainers. Sandra Conti, MS, received her master’s degree in clinical psychology from Benedictine University. Conti was formerly a substance abuse counselor at Gateway Foundation’s Aurora, IL, and she is currently working with Guided Path Psychological Services in Palatine, IL, where she provides individual and group counseling for clients with mental health issues and substance abuse problems.

The MBS model presented by the authors is aimed to help clients with addictions to achieve sobriety through the enhancement of awareness, the acceptance of the experiences and the clarification of values. It is based on three evidence-based programs – all of them registered in the National Registry of Evidence-Based Programs and Practices – moreover, there is a separately conducted pilot study for both residential and intensive outpatient (IOP) care. According to this study, 63.4% of the clients successfully completed treatment compared to an average statewide rate (49.2%) for IOP patients.

The book consists of two parts. The first part explains the therapeutic foundation and approach thoroughly and the second part contains the detailed manuals for two levels of care: for IOP care and for residential treatment. Several appendices provide important material for the treatment sessions and a list of publications connected to the main topics of the book.

Professionals can familiarize themselves with the basics of the three contributing models in Chapter 1. Regarding ACT we can read about the six core processes of psychological rigidity which can lead to more suffering and less engagement in life. We can also learn about the opposite – psychological flexibility –, which may lead to a fully lived life. The essential aspects of MI – partnership, acceptance, compassion and evocation – and its use in practice are introduced as well. Regarding RPT the analysis of high-risk situations, cognitive restructuring and lapse management among other useful aspects are highlighted.

In Chapter 2 the adaptation of MBS in different levels of care are introduced. Though criteria of patient placement can differ from country to country, the presentation of the six placement criteria of ASAM PPC-2R (American Society of Addiction Medicine Patient Placement Criteria for the Treatment of Substance-Related Disorders) can help every practitioner to reach the highest effectiveness through treatment planning.

Some important therapeutic principles – such as the flexibility of focus and the personalization of the approach – are emphasized in Chapter 3 and there are useful tips for lapse management as well. This chapter highlights one of the greatest virtues of the concept: the MBS was designed for open-groups. This is very helpful for practitioners who often face the challenge of scheduling and maintaining a closed group in real life. The open-group format has several advantages including cost-effectiveness and easier scheduling. In this group format, new members can learn from the experiences of the “older” members and inversely, the more experienced members can see their progress. For clients it is easier to join an open-group, when they are ready to change, and their motivation is less likely to vanish while they are waiting for a closed-group to begin.

In Chapter 4 the MBS curriculum for IOP patients is presented. This chapter contains detailed information for group and session arrangement issues (e.g. length, parts of the sessions, tools, handouts, etc.). Ten sessions with different core topics are introduced in details; the sessions are dealing with important therapeutic issues such as defusing from addiction, value-based avoidance or relationships. Because of the open-group format the topics are rotated. There are repeated parts of the sessions, including the brief review of the MBS-model, which can deepen the perspectives and enhance skills, but it is important for clinicians to be cautious and keep the redundancy at minimum to maintain the participants’ involvement.

The curriculum for residential care in Chapter 5 is as thorough as the curriculum for IOP patients. The authors emphasize the main purposes (e.g. motivation enhancement, planning) for this level of care and organize the sessions accordingly. There are twelve rotated topics for residential treatment, which are placed at the third part of a 3-hour session and which are less underlined than in the IOP curriculum. The three parts of the sessions include a two-tiered check-in, an exercise, called “important things” which concerns and unfolds important life domains and the specific topic of the day such as relapse prevention, which is the core topic of the first three subsequent sessions, role playing and drug refusal skill development – which are also recurrent topics – or spirituality which appears in session 8.

Given that the curricula is developed for open-groups the book itself is appropriate for the “open group” of readers as well. It has the same advantages as the open-group format for the patients, because every reader has different amount of knowledge and experience with mindfulness techniques, some are experts of the field and some are only in the beginning of exploring them. Though the book’s main aim is to provide a useful manual for clinicians, it can be informative for different kinds of readers.

Some challenges are still open for clinicians who will apply MBS in the treatment of addictions. One of this is the closure of the treatment for each client, which is an important task for professionals and can be crucial regarding the client’s further progress. Another topic for the future investigations is the application of MBS for clients with behavioral addictions to broaden the scope of this promising new therapeutic method.

