# Supplementary material for: Book Reviews
Source: J Behav Addict. 2015 Mar 18;4(1):32–4. doi: 10.1556/JBA.4.2015.1.8 (PMC4394852; doi:10.1556/JBA.4.2015.1.8)
Supplement: Related texts

# Book review

**Published:** 2015-03-18

**Authors:** Attila Körmendi

**Affiliations:** Department of Personality and Clinical Psychology Institute of Psychology, University of DebrecenDebrecenHungarykormendi.ati@freemail.hu

*Internet gambling* by Sally Gainsbury provides a comprehensive and systematic overview of online gambling. Given the recent technological advancements and easy access to Internet this topic is current and very important. The volume contains nine chapters each of which deals with Internet gambling from a different perspective. There are chapters for clinical psychologists that describe individual characteristics of online gamblers within vulnerable populations. Other chapters approach online gambling from a wider perspective providing a market overview or detailed policy recommendations. We may also read about gambling sites’ marketing strategies to acquit and retain customers.

Chapter 1 serves as an introduction to the topic; major historical milestones are described from the appearance of the first authentic gambling site in 1993 to present day. Online gambling is constantly changing including the type of games, the market and the policies, therefore it is challenging to generalize previous results to the current situation. Market-driving trends such as new channels for Internet, easy access, liberalized regulation, etc. are detailed. It appears that these drivers are effective and the market grows and expands to new areas as we can read in Chapter 2, which is a systematic market overview. As far as the product market is concerned: wagering (39%) leads the global Internet gambling market followed by casino (26%). Regarding regional market overview, Europe is the leading market with 47% of total market share. Prevalence from more than fifteen countries ranges between 1 and 13%. Furthermore, there is a large competition among gambling sites to acquit and retain customers. As we will see later, this is one of the most important factors that determines whether an online gambling site is successful or not. The chapter deals with payment methods (there are more than two-hundred) and the different types of gambling sites. One interesting example is the “practice sites” that offer free gambling-type games with the intention to introduce newbies to the world of Internet gambling.

Chapter 3 is an analysis of policy options outcomes and consequences. Policies differ by country. There are countries where it is prohibited for banks and credit card companies to process transactions from Internet gambling sites, but blocking access to gambling sites is also an option. Due to the anonymous nature of online gambling, upholding laws can be difficult sometimes. Legalization is another controversial question. Legalization of online gambling results in tax revenue from the gaming operator, which protects the consumer but may contribute to the growth of online gambling. In addition, legislation may have additional social costs, although currently there are insufficient studies to assess the social impact of online gambling. Government ownership could be a possible solution for this issue. For example in Norway and in Sweden single sites are run by the government. The rest of the chapter deals with jurisdictions and their impact on Internet gambling in more than fifteen countries.

The next chapter describes the individual characteristics of Internet gamblers. Online gamblers seem to be different from traditional gamblers: they have different motivational background, report fewer physiological effects and gamble less for emotional reasons compared to traditional gamblers. Gender, age and social demographic characteristics are also discussed. As for motivation there are specific counter-motivations, coming from perceived disadvantages of Internet gambling, which are not of concern to traditional gamblers. Perceived disadvantages may include non-paying or cheating of gambling sites or other players’ possibility to cheat. As described later in the volume, these fears may have their roots in reality. For example an independent player protection organization (eCOGRA) reported that one-third of survey respondents have had issues with a gambling site. Many cited research papers state that trust in a gambling site is the most important factor which determines whether or not a gambler is willing to play online. This perspective is helpful for clinicians to understand their clients’ gambling career and the development of addiction.

There are numerous risks in gambling as we can read in Chapter 5. Online sites are often operated illegally and registered players have to face several risks. For example, there exists a possibility that online gambling sites would declare bankruptcy and site members’ winnings are not paid out. Furthermore, personal details of player accounts may be sold to call centers, which constitutes an identity theft. Examples of unfair player practices are introduced from the manipulation of gaming software to layer collusion, a covert co-operation between gamers.

Chapter 6 outlines the vulnerable populations of online gamblers. One such population is the adolescents (and young adults) because they are familiar with Internet technology and play gambling online at higher rates than adults. Another vulnerable population can be the problem gamblers, who are present in higher rates among online gamblers than non-problematic gamblers. The factors which make the online environment more attractive to traditional problematic gamblers are summarised in this chapter. These factors include for example the high availability, accessibility and anonymity of online gambling games when traditional gambling games are not available for them. Online gambling games can be played at a higher speed, bets can be placed more quickly and eventually the game provides higher event frequency for them which is probably highly attractive for traditional gamblers (especially impulsive ones). Another main theme of this chapter is the advertising restrictions. Advertisements often mislead gamblers about the chances of winning and the size of gains. A good example is a banned gambling advertisement (targeted towards adolescents), which claimed that online wagering improves sexual prowess and self-esteem.

Chapter 7 deals with responsible gambling strategies used by gambling sites in order to protect their consumers. These harm-minimalizing strategies can prevent gambling problems but more studies are needed to assess their effectiveness. Such a strategy is for example player identification to prevent underage play or duplicate accounts. Or, there may be time delays between rounds to prevent chasing. During game sessions pop-up messages raise players’ awareness of their behavior by informing them about play time or losses. Messages may appear on screens about statistical probabilities of current game winning. These strategies are somehow similar to those used in cognitive behavior therapies of problem gambling. Players receive feedback about their gambling behavior and therefore cognitive distortions are likely to be reduced via the support of logical thinking.

Chapter 8 is about the future trends of online gambling. According to the author, Internet gambling will continue to evolve, as new traditional gambling and non-gambling companies enter the online gambling market. There will be a natural selection among gambling sites as they compete with each other for customers and eventually the market will spread towards India and Asia. Smart phones and other devices, which are able to connect to the Internet will also increase market growth. Finally, Chapter 9 summarizes previous chapters and describes problems of online gambling studies. To facilitate future researches of online gambling final thoughts are given about the gaps in Internet gambling research. Due to its comprehensive nature and its easy-to-read style this book is recommended to anyone who intends to engage in online gambling research or wishes to expand their knowledge about this behavior.
